# Ubiquitin-specific protease 14 targets PFKL-mediated glycolysis to promote the proliferation and migration of oral squamous cell carcinoma

**DOI:** 10.1186/s12967-024-04943-z

**Published:** 2024-02-22

**Authors:** Xingming Zhang, Lou Geng, Yi Tang, Yingying Wang, Youping Zhang, Chujiao Zhu, Hu Lei, Hanzhang Xu, Qi Zhu, Yingli Wu, Wenli Gu

**Affiliations:** 1 Department of Clinical Laboratory, Shanghai Ninth People’s Hospital, Shanghai Jiao Tong University School of Medicine, Shanghai, 200011 China; 2 Hongqiao International Institute of Medicine, Shanghai Tongren Hospital/Faculty of Basic Medicine, Key Laboratory of Cell Differentiation and Apoptosis of the Chinese Ministry of Education, Shanghai Jiao Tong University School of Medicine, Shanghai, 200025 China; 3 Department of Hematology, Shanghai Ninth People’s Hospital, Shanghai Jiao Tong University School of Medicine, Shanghai, 200025 China; 4 Research Units of Stress and Tumor (2019RU043), Chinese Academy of Medical Sciences, School of Medicine, Shanghai Jiao Tong University, Shanghai, 200025 China

**Keywords:** USP14, Deubiquitination, PFKL, Glycolysis, OSCC

## Abstract

Aberrant upregulation of the ubiquitin-specific protease 14 (USP14) has been found in some malignant tumors, including oral squamous cell carcinoma (OSCC). In this study, we further demonstrated that aberrantly overexpressed USP14 was also closely related to adverse clinicopathological features and poor prognosis in patients with OSCC, so we hypothesized that USP14 might act as a tumor-promoting factor during the progression of OSCC. Notably, we originally proved that USP14 is a deubiquitinating enzyme for phosphofructokinase-1 liver type (PFKL), a key rate-limiting enzyme involved in the glycolytic pathway. USP14 interacts with PFKL and enhances its stability through deubiquitination in OSCC cells, which in turn enhances PFKL-mediated glycolytic metabolism and ultimately promote cellular proliferation, migration, and tumorigenesis. In this work, we have also demonstrated for the first time that USP14 is a critical regulator of glycolysis in OSCC and verified a novel mechanism whereby it is involved in tumor metastasis and growth. Collectively, our findings provide novel insights into the tumor-promoting role of USP14 and establish mechanistic foundations for USP14-targeting therapies.

**Supplementary Information:**

The online version contains supplementary material available at 10.1186/s12967-024-04943-z.

## Introduction

Oral squamous cell carcinoma (OSCC) is the most prevalent oral malignancy, accounting for 80–90% of all malignant neoplasms of the oral cavity [1]. Although there are currently a number of treatments for OSCC in clinical use, its prognosis remains relatively poor [2]. At present, surgical resection remains the preferred treatment for OSCC. However, postoperative dysfunctions in chewing, swallowing, and speech can significantly affect the quality of life of patients treated surgically for OSCC [3]. Therefore, the occurrence and development mechanism behind OSCC remains a very challenging field in the research of head and neck malignancies. As a result, many efforts have been made to identify novel and effective treatment strategies to improve the prognosis of OSCC.

Aberrant glucose metabolism is an emerging hallmark of cancer [4]. Cancer cells significantly increase their glucose uptakes and preferentially convert glucose into lactate, even under aerobic conditions. This is known as the Warburg effect, or aerobic glycolysis [5]. Aerobic glycolysis confers a selective advantage in cancer cells, allowing them to meet their high energy and biosynthetic demands in the unique tumor microenvironment, which is of great significance to the occurrence and progression of tumors. As a result, tumors are also regarded as a metabolic disease [6]. Based on the aberrant metabolic characteristics of cancer cells, selectively targeting key enzymes involved in the glycolytic pathway may provide an effective therapeutic strategy to inhibit the proliferation of cancer cells.

Phosphofructokinase-1 (PFK-1) catalyzes the irreversible conversion of D-fructose 6-phosphate (F6P) to fructose 1,6-bisphosphate (F-1,6-BP). This conversion represents the first committed step of glycolysis [7]. Phosphofructokinase includes three isozymes in humans: liver (L), muscle (M), and platelet (P) [8]. As a key rate-limiting enzyme involved in the glycolytic pathway, the activity of PFK-1 is modulated by allosteric regulation and post-translational modifications, including glycosylation, helping it fine-tune the metabolic balance [9, 10]. In addition, it has been reported that the expression of the liver isozyme of PFK-1 (PFKL) is also regulated by ubiquitination, which is mediated by the E3 ligase A20 [11]. Studies have demonstrated that the expression of PFK-1 in cancer cells is often markedly elevated and that the compositions of PFK isozymes are also frequently altered with PFKL and PFKP typically being expressed at higher levels in cancer cells compared with PFKM [12]. Nevertheless, the exact expression patterns of PFK-1 in OSCC and the corresponding regulatory mechanisms behind these patterns remain largely unknown.

Ubiquitin-specific protease 14 (USP14) is one of the major proteasome-associated deubiquitinating enzymes that reversibly associates with the proteasomal 19 S regulatory particle. USP14 recognizes and removes ubiquitin chains from its ubiquitinated substrates, thus rescuing proteins from degradation in the proteasome. This process is critical to maintaining proper protein homeostasis [13]. USP14 has a wide range of biological functions and plays essential roles in cellular signaling, inflammatory responses, neurological functions, and tumorigenesis [14]. The coding gene for USP14 is located on human chromosome 18p11.32, and studies have found that this region is closely related to the regulation of human malignant tumors [15]. We previously reported that USP14 was significantly overexpressed in OSCC and that knockdown of USP14 markedly inhibited tumor growth and triggered apoptosis in cancer cells [16].

In this study, we further demonstrated that aberrantly overexpressed USP14 is also closely related to the poor prognosis of OSCC. We therefore hypothesized that USP14 might act as a tumor-promoting factor during the progression of OSCC. Notably, we demonstrated that USP14 is a deubiquitinating enzyme for PFKL, a key rate-limiting enzyme involved in the glycolytic pathway in certain cases of OSCC. USP14 interacted with PFKL, stabilized and slowed down its degradation through deubiquitination, and thus influenced the proliferation, migration and glycolysis rates of OSCC cell lines. Thus, we confirmed for the first time in the literature (to the best of our knowledge) that USP14 is a critical regulator of glycolysis in OSCC. We also verified a novel mechanism for USP14’s involvement in tumor growth and metastasis via its regulation of PFKL. Given that aberrant glucose metabolism is linked to OSCC, this study may highlight novel potential therapeutic targets for patients with OSCC.

## Materials and methods

### Patients and tissue samples

A total of 70 tumor tissue specimens were collected during surgical resection from patients with primary OSCC being treated at the Department of Oral and Maxillofacial Surgery, Shanghai Ninth People’s Hospital, Shanghai Jiao Tong University School of Medicine (Shanghai, China) between 2019 and 2022. All of the patients provided written informed consent for their specimens to be used for this study. None of the patients had received chemotherapy and/or radiotherapy prior to their surgeries. Adjacent normal oral epithelial tissues were obtained from points at least 1 cm away from the tumor borders and confirmed to be free of tumor cells via microscopy. The resected tissues were fixed with 4% paraformaldehyde and embedded in paraffin for immunohistochemical staining. Some collected tissues (n = 8) were stored in liquid nitrogen for Western blotting analysis. All study protocols were approved by the review board of the hospital’s ethics committee.

### Bioinformatic analysis

We analyzed RNA-seq expression data of patients with OSCC pulled from The Cancer Genome Atlas (TCGA). Gene expression analysis was conducted and visualized using the “ggplot2” package for R, after data normalization. Using quartile values of USP14 expression as cutoffs, the patients were divided into USP14-high and USP14-low groups. The “survival” R package was used to evaluate the correlation of USP14 expression with overall survival (OS), progression free interval (PFI) and disease- specific survival (DSS). Hazard ratios (HRs) and 95% confidence intervals were also calculated.

### Plasmids, reagents and antibodies

To construct a plasmid for USP14 overexpression, we inserted the sequence of *USP14* into the pBABE plasmid, along with HA and Myc tags. The flag-tagged PFKL gene was also cloned into a pCDH-CMV-MCS vector. A small-molecule inhibitor of USP14, b-AP15 (cat. no. S4920; Selleck Chemicals, Inc., Houston, TX, USA), was used at a final concentration of 1 μM for all experiments. MG132 (cat. no. M7449; Sigma-Aldrich, Inc., Saint Louis, MO, USA) was used at a final concentration of 5 μM. Translational inhibitor cycloheximide (cat. no. S7418; Selleck Chemicals, Inc., Houston, TX, USA) was used at a final concentration of 10 μg/mL. The glucose analog and competitive inhibitor of glucose metabolism 2-deoxy-d-glucose (cat. no. HY-13966; MedChemExpress, Monmouth Junction, NJ, USA) was used at a final concentration of 5 mM. Dimethyl sulfoxide (DMSO) purchased from Sigma-Aldrich was used as a solvent and negative control. Primary antibodies against the following proteins were used for Western blotting analysis: USP14 (cat. no. sc-398009; 1:1000 dilution; Santa Cruz Biotechnology, Inc., Dallas, TX, USA), Flag (cat. no. 20543-1-AP; 1:2000 dilution; ProteinTech Group, Inc., Chicago, Illinois, USA), HA (cat. no. #2367; 1:1000 dilution; Cell Signaling Technology, Inc., Boston, MA, USA), Ub (cat. no. #3936; 1:1000 dilution; Cell Signaling Technology, Inc., Boston, MA, USA), PFKL (cat. no. ab97443; 1:2000 dilution; Abcam, PLC., Cambridge, UK), PFKP (cat. no. sc-514824; 1:1000 dilution; Santa Cruz Biotechnology, Inc., Dallas, TX, USA), PFK-1 (cat. no. sc-166722; 1:1000 dilution; Santa Cruz Biotechnology, Inc., Dallas, TX, USA) and β-actin (cat. no. HRP-60008; 1:5000 dilution; ProteinTech Group, Inc., Chicago, IL, USA). HRP-conjugated anti-rabbit or anti-mouse IgG secondary antibodies (cat. no. AS014 or AS003; 1:10,000 dilution; ABclonal Biotech Co., Ltd., Wuhan, Hubei, China) were used to detect the primary antibodies.

### Cell culture

Normal human oral keratinocytes (HOKs) and OSCC cell lines (HN4, HN6, HN30 and Cal-27) were provided by Shanghai Key Laboratory of Stomatology, Shanghai Ninth People’s Hospital, Shanghai Jiao Tong University School of Medicine (Shanghai, China). The cells were cultured in Dulbecco’s Modified Eagle’s Medium (DMEM; Hyclone, Inc., Logan, UT, USA) containing 10% fetal bovine serum (FBS; Gibco BRL, Inc., Gaithersburg, MA, USA), 100 units/mL penicillin, and 100 μg/mL streptomycin, at 37 °C in a humidified atmosphere containing 5% CO_2_. All cells used in the study were regularly treated with mycoplasma removing agent (New Cell & Molecular Biotech, Suzhou, Jiangsu, China), and mycoplasma contamination was detected using a MycoSEQ™ Mycoplasma Detection Kit (cat. no. 4460623; Thermo Fisher Scientific, Inc., Waltham, MA, USA).

### Lentivirus and retroviral transfection

A short hairpin RNA (shRNA) clone and compatible packaging plasmids (pGag/Pol and pVSV-G; pMD2.G and psPAX2) were co-transfected into HEK293T cells for 48 h, after which the supernatant containing the viral particles was collected by filtration (0.45-μm cellulose acetate filter; Millipore Sigma, Merck KGaA, Darmstadt, Germany). HN4 and HN6 cells were incubated with the virus-containing supernatant and supplemented with 8 μg/mL polybrene (Beyotime Institute of Biotechnology, Haimen, Jiangsu, China) for 6 h and then replaced with fresh complete medium. After 48 h, puromycin (Beyotime Institute of Biotechnology, Haimen, Jiangsu, China) was added at a final concentration of 2 μg/mL, to screen for positive cells. The targeting sequences of the shRNA were shown to be the following: shUSP14-1#, 5′-TCAAGGATCTAGAAGATAA-3′ (sense) and 5′-TTATCTTCTAGATCCTTGA-3′ (antisense); shUSP14-2#, 5′-AGATGTTTACTGCACACCT-3′ (sense) and 5′-AGGTGTGCAGTAAACATCT-3′ (antisense); shPFKL-1#, 5′-CTGAAGATGCTGGCACAATAC-3′ (sense) and 5′-GTATTGTGCCAGCATCTTCAG-3′ (antisense); shPFKL-2#, 5′-GCTCCATCGATAACGACTTCT-3′ (sense) and 5′-AGAAGTCGTTATCGATGGAGC-3′ (antisense).

### RNA extraction, reverse transcription and quantitative real-time polymerase chain reaction

TRIzol® reagent (Thermo Fisher Scientific) was used to extract total RNA from OSCC cells according to the manufacturer’s instructions, and cDNA was synthesized using a High-Capacity cDNA Reverse Transcription Kit (Thermo Fisher Scientific) in a volume of 20 μL. The ABI 7500 Real-Time PCR system (Applied Biosystems, Inc. Foster City, CA, USA) was used to perform reverse transcription-quantitative real-time polymerase chain reaction (RT-qPCR) using SYBR Green qPCR Master Mix (Takara Biotechnology, Inc., Dalian, Japan). The reaction mixture in each well contained a final volume of 10 μL based on 5 ml of SYBR Green qPCR Master Mix reagent, 10 μM of each pair of primers, and 50 ng of cDNA. The qPCR cycling was performed according to a previously described protocol [17]. The relative quantification of the target gene was calculated using the 2^-ΔΔCt^ method, normalized to β-actin gene expression. The primer sequences we used were as follows: USP14 forward 5′-GGGAAATGGCTTCAGCGCAGTA-3′ and USP14 reverse 5′-CACCTTTCTCGGCAAACTGTGG-3′; PFKL forward 5′-AAGAAGTAGGCTGGCACGACGT-3′ and PFKL reverse 5′-GCGGATGTTCTCCACAATGGAC-3′; ACTB forward 5′-CATGTACGTTGCTATCCAGGC-3′ and ACTB reverse 5′-CTCCTTAATGTCACGCACGAT-3′.

### Immunoprecipitation and Western blotting analysis

For co-immunoprecipitation assays, whole cell lysates were obtained using lysis buffer (1% NP-40; 50 mM Tris–HCl, pH 7.4; 150 mM NaCl; 5 mM EDTA; 0.02% SDS) supplemented with a 1 × protease inhibitor cocktail (Beyotime Biotechnology Co., Ltd. Shanghai, China) prior to lysis on a rotary shaker at 4 °C for 30 min. The cell lysates were then centrifuged at 12,000 rpm for 20 min at 4 °C. After centrifugation, the supernatant was collected and incubated with indicated primary antibodies or IgG control overnight at 4 °C. The following day, protein A/G agarose beads (cat. no. sc-2003; Santa Cruz Biotechnology) were added for additional 2–4 h. Following this incubation, the beads that were bound to antigen–antibody immunocomplexes were retained and washed with phosphate buffered saline (PBS) for three times. The samples were then analyzed by Western blotting.

For Western blotting analysis, the cells were collected and lysed with lysis buffer (50 mM Tris–HCl, pH 6.8; 100 mM DTT; 2% SDS; 10% glycerol). The collected protein samples were quantitated using a bicinchoninic acid (BCA) assay reagent (cat. no. 23227; Thermo Fisher Scientific), and equal amounts (30 µg/lane) of total protein were loaded on 8–12% SDS gels to be separated by electrophoresis and transferred onto a nitrocellulose membrane (Millipore Sigma; Merck KGaA). The membranes were blocked with 5% skimmed milk in PBS with 0.1% Tween 20 (PBST) for 1 h at room temperature (RT) and then washed twice with PBST. The membranes were incubated with primary antibodies overnight at 4 °C, followed by incubation with horse radish peroxidase (HRP)-conjugated anti-rabbit or anti-mouse IgG secondary antibody for 1 h at RT. Finally, the signals were analyzed using a chemiluminescence phototope-HRP kit (Thermo Fisher Scientific), according to the manufacturer’s instructions.

### Mass spectrometry analysis

To identify potential USP14-binding proteins, we established HN4 cells that stably overexpressed retrovirally-mediated and HA-tagged USP14. Lysates from these cells were immunoprecipitated with anti-HA or anti-Mouse IgG antibodies as negative controls. The samples were subjected to electrophoresis and silver staining (Beyotime Institute of Biotechnology) according to the manufacturer’s instructions. Proteins in the silver-stained regions were then identified using mass spectrometry analysis, according to a previously reported protocol [18]. Briefly, each lane was cut into small squares of 1 mm × 1 mm. Then, the proteins identified as stained bands in the gels were subjected to in situ digestion using trypsin (trypsin: protein = 1:50 w/w) at 37 °C for 16 h, according to a previously reported protocol [19]. The peptides were recovered by extraction with 50% acetonitrile (ACN) and 0.1% trifluoroacetic acid (TFA) at 37 °C for 30 min (2–3 times). The peptides were lyophilized and analyzed by an EASY-nLC 1000 system connected to a Q Exactive mass spectrometer and an Orbitrap Fusion mass spectrometer (Thermo Fisher Scientific). The results were searched using MASCOT software, with search parameters that are listed in Additional file 5: Table S1.

### Immunofluorescence staining

Briefly, 2 × 10^5^ HN6 cells were seeded in confocal dishes until optimal cell density was reached. They were fixed with 4% paraformaldehyde for 20 min and permeabilized with 0.5% Triton X-100 for 15 min at RT. Further, cells were blocked with 1% bovine serum albumin (BSA) in PBS for 1 h at RT. Each step was followed by a 10–15 min wash using PBS. Primary antibodies against USP14 (cat. no. sc-398009; 1:50 dilution; Santa Cruz Biotechnology) and PFKL (cat. no. ab97443; 1:100 dilution; Abcam) were then incubated with cells overnight at 4 °C for co-staining. The next day, after washing three times with PBS, goat anti-mouse IgG, IgM (H + L) secondary antibody, Alexa Fluor™ 488 (cat. no. A10680; 1:1000 dilution; Thermo Fisher Scientific) and goat anti-rabbit IgG (H + L) highly cross-adsorbed secondary antibody, Alexa Fluor™ Plus 555 (cat. no. A32732; 1:1000 dilution; Thermo Fisher Scientific) were applied to the sections for 1 h at RT. The nuclei were counterstained with 4′,6-diamidino-2-phenylindole (DAPI) and the final fluorescence imaging was performed using a laser confocal microscope (Nikon, Nagoya, Japan).

### Immunohistochemistry and semi-quantitative analysis of USP14

Immunohistochemical staining was performed on paraffin-embedded, 5-μm-thick tumor tissue sections according to standard procedures. Briefly, the slides were deparaffinized, rehydrated, and the antigens were retrieved by heating in a microwave oven at 100 °C for 15 min using 10 mM sodium citrate buffer (pH 6.0). The slides were then blocked with 5% normal goat serum for 30 min at RT and incubated with mouse anti-human USP14 antibody (cat. no. sc-398009; 1:200 dilution; Santa Cruz Biotechnology) overnight at 4 °C. After a 1-h incubation with a HRP-conjugated secondary antibody (cat. no. abs996; Absin) at RT, the slides were visualized with diaminobenzidine (DAB) and counterstained with hematoxylin.

A semi-quantitative analysis of USP14 was performed by analyzing the percentage of positive cells and staining intensity in a total of five random low-power fields per sample. Immunoreactive score (IRS) was determined by the product of the staining proportion score (PS) and staining intensity score (IS). Specifically, PS was defined as 0 (0%), 1 (1–25%), 2 (26–50%), 3 (51–75%), or 4 (76–100%); IS was classified as 0 (negative, no brown particle staining), 1 (weak, light brown particle), 2 (moderate, moderate brown particle), or 3 (strong, dark brown particle). Based on the average of five low power fields IRS scores, the samples were divided into two groups: low USP14 expression (IRS ≤ 3) and high USP14 expression (IRS > 3).

### In vitro ubiquitination assays

To assess in vitro ubiquitination, HEK293T cells were co-transfected with Flag-tagged PFKL and HA-tagged ubiquitin and then treated with MG132 (5 μM) for 5 h before collecting. The immunoprecipitates were obtained by incubating the cell lysates with anti-Flag antibody and protein A/G agarose beads under denaturing conditions (50 mM Tris- HCl, pH 8.0; 50 mM NaCl; 10 mM DTT; 1 mM EDTA and 5% glycerol) overnight at 4 °C. The PFKL ubiquitination level was then visualized by Western blotting analysis.

### Cell proliferation analysis

Cell proliferation was examined using a Cell Counting Kit-8 (CCK-8) assay kit (CK04; Dojindo Molecular Technologies, Inc., Kumamoto, Japan) according to the manufacturer’s instructions. Briefly, OSCC cells (5 × 10^3^ cells/well) were seeded in 96-well plates for 4 days. To determine the viability of the cells, a 10 μL aliquot of CCK-8 solution was added in each well for an additional 4 h incubation period. Optical density (OD) at 450 nm was then detected using a Synergy H4 Hybrid Microplate Reader (BioTek Instruments, Inc., Winooski, VT, USA).

### Wound healing assay

Cells were infected with lentivirus as described above and seeded in 6-well plates at a density of 5 × 10^5^ cells per well in 2 mL complete DMEM. Once the cells reached 100% confluence, a single scratch wound of the cellular monolayer was made using a 10-µL plastic pipette tip. The cells were washed twice with PBS and incubated with fresh and serum-free DEME at 37 °C in a humidified atmosphere containing 5% CO_2_. Images were obtained at this point, as well as 24 h later, to compare the cellular migration as it closed the scratch wound. The cell-free areas in multiple fields were observed using a service provided by Wimasis, which permitted the images to be uploaded online to be analyzed, before being sent back to our server [20].

### Transwell assay

A cell migration assay was performed using 24-well Transwell cell culture plates (Corning Inc., Corning, NY, USA) with a polycarbonate membrane (pore size = 8 μm) at the bottom of the upper chamber. Cells were seeded in the upper chamber, where they cultured with 200 μL of medium that didn’t contain FBS, while 700 μL of medium containing 20% FBS was added to the lower chamber. At the indicated time points, the non-migrated cells on the upper surface were removed by wiping with a cotton swab, and the migrated cells on the lower surface were fixed with 4% paraformaldehyde for 15 min, dried in a ventilated place, and stained with 0.1% crystal violet for 30 min. Five fields under the microscope (Olympus, Tokyo, Japan) were selected at random and the number of cells in each field was counted. This counting was repeated in triplicate to obtain reliable cell counts.

### Glucose uptake and lactate detection assay

Cellular glucose uptake was measured according to the manufacturer’s instructions (cat. no. ab136956; Abcam, PLC., Cambridge, UK). Briefly, OSCC cells were transfected with pLKO.1-shPFKL, pGIPZ-shUSP14, or control vector plasmids. A total of 1 × 10^4^ cells/well were seeded in a 96‐well culture plate and kept in serum-free medium to increase glucose uptake. After 24 h, cells were incubated in 10 μL/well of 10 mM 2-DG at 37 ℃ for 20 min. They were then exposed to 90 μL/well extraction buffer to lyse the cells. Following this treatment, we froze/thawed the lysates once and heated samples at 85 °C for 40 min. Next, we cooled the cell lysates on ice for 5 min and neutralized by adding 10 µL of neutralization buffer. Samples or standards were incubated with reaction mix at 37 ℃ for 40 min. At the end of the experiment, the absorbance ratio increase at 535/587 nm was assessed using a Synergy H4 Hybrid Microplate Reader (BioTek Instruments).

Lactate production was analyzed using a lactate colorimetric assay kit, according to the manufacturer’s instructions (Njjcbio, Nanjing, Jiangsu, China). Briefly, the cells were transfected with the previously described plasmids. After 24 h, the supernatants were collected and incubated with 100 μL/well of enzyme working solution as well as 20 μL/well of chromogenic agent, at 37 °C for 10 min. Stopping solution (200 μL) was then added to each sample. The absorbance was measured using a Synergy H4 Hybrid Microplate Reader (BioTek Instruments) at 530 nm. Lactate production levels were calculated based on the known lactate concentration of standards and normalized to the total protein abundance.

### In vivo experiments

Male BALB/c nude mice (6 weeks of age, weight 17–19 g) were purchased from Shanghai SLAC Laboratory Animal Co., Ltd. (Shanghai, China). All experimental procedures performed for this study were approved by the Laboratory Animal Care and Use Committees of the Shanghai Jiao Tong University School of Medicine (Shanghai, China). Briefly, the BALB/c nude mice were randomly assigned to three groups. All had a total of 2 × 10^6^ HN6 cells stably transfected with either shCtrl, shUSP14-1#, or shUSP14-1# + PFKL. The cells were suspended in 100 μL of PBS and injected subcutaneously into the right flanks of the mice. When the resultant tumors became palpable, their sizes were monitored every 2 days and the tumor volumes were calculated using the standard formula: width^2^ × length/2. On day 28, the tumor-bearing mice were sacrificed, and their tumors were immediately excised and fixed in 4% phosphate-buffered paraformaldehyde for 24 h at 4 °C, prior to being embedded in paraffin for further study.

### Statistical analysis

All statistical analyses were performed using GraphPad Prism 8.0.2 software and R version 3.6.1. The χ^2^ or Fisher’s exact test was used to evaluate correlations between USP14 expression and clinicopathological features, as well as the statistical significance of the animal experiments. OS, PFI, and DSS rates were estimated using the Kaplan–Meier method. For in vitro experiments, the significance of the results was determined using a nonparametric 2-tailed Student’s t-test. *P* values < 0.05 were considered statistically significant.

## Results

### Aberrant upregulation of USP14 is associated with poor clinical outcomes in oral squamous cell carcinoma

**Fig. 1:**
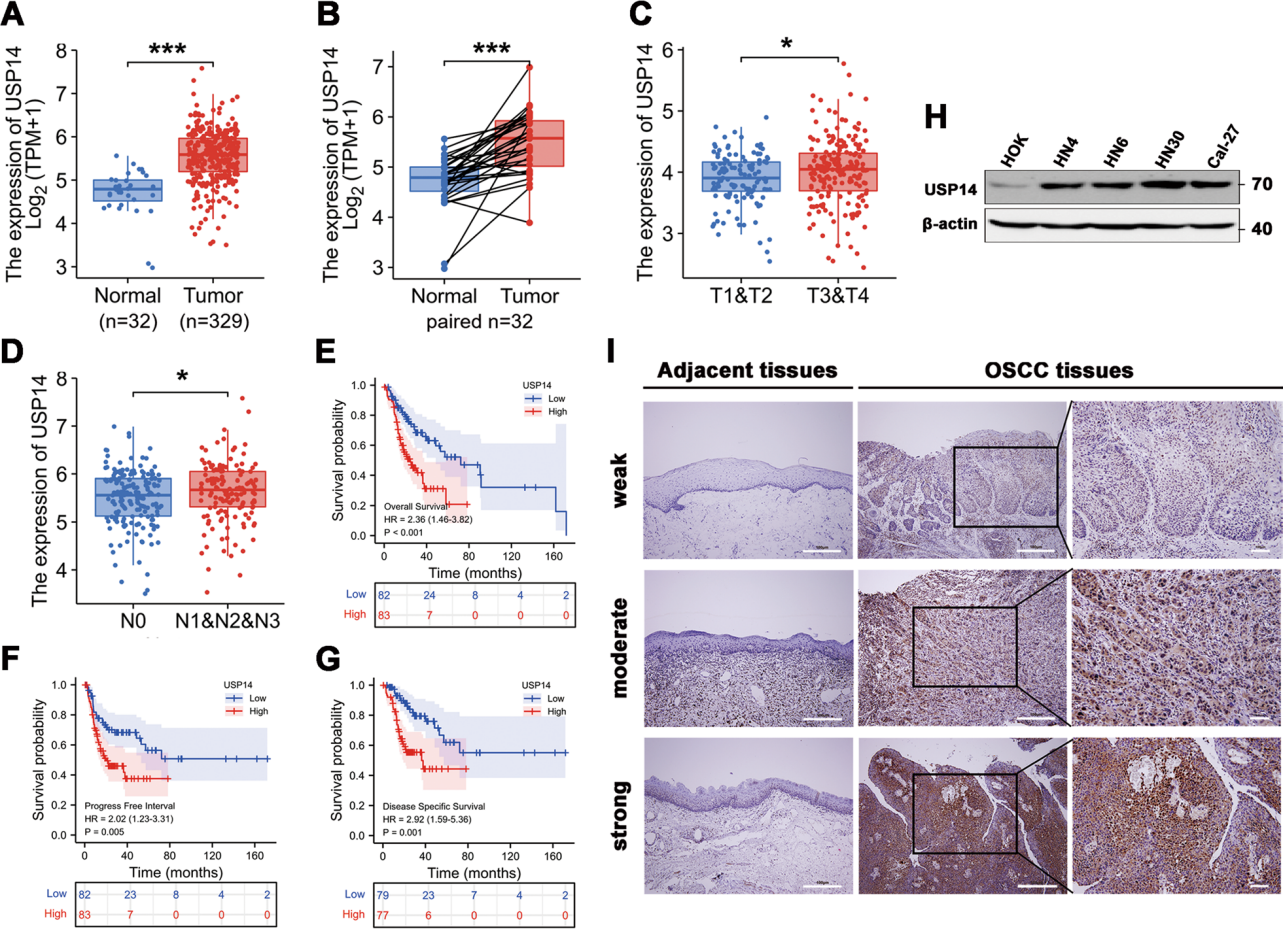
The upregulation of ubiquitin-specific protease 14 predicts poor clinical outcomes in patients with oral squamous cell carcinoma.** A–D** USP14 expression of patients with OSCC from the TCGA database. USP14 was aberrantly overexpressed in OSCC tumor tissues compared to normal tissues (**A**). USP14 expression in OSCC was higher than that in matched normal samples (**B**). Upregulated expression of USP14 was significantly associated with T stage (**C**) and N stage (**D**). **E–G** Overall survival (OS), progress free interval (PFI) and disease specific survival (DSS) based on USP14 expression in OSCC (TCGA). USP14 high (red) group corresponds to the fourth quartile of expression, while USP14 low (blue) group corresponds to the first quartile. **H** The protein levels of USP14 in four OSCC cell lines were compared with a normal oral epithelial cell line HOK by Western blotting analysis. **I** The expression pattern of USP14 was examined in OSCC tissues and their adjacent normal epithelial tissues by immunohistochemical staining (n = 70). *P < 0.05, ***P < 0.001

We analyzed OSCC transcriptome sequencing datasets from the TCGA database and evaluated the correlation between the mRNA expression levels of USP14 and clinicopathological features of the disease. Our results indicated that USP14 was aberrantly overexpressed in OSCC tumor tissues compared to normal ones (Fig. 1A, B). We also observed that the expression of USP14 was correlated with certain clinicopathological features in patients with OSCC. As shown in Fig. 1C, D, upregulated expression of USP14 was significantly associated with T stage (*P* < 0.05) and N stage (*P* < 0.05). Survival curves also demonstrated that a higher transcriptional level of USP14 was significantly correlated with shorter OS (HR = 2.36, *P* < 0.001), PFI (HR = 2.02, *P* = 0.005) and DSS (HR = 2.92,* P* = 0.001) in patients with OSCC (Fig. 1E–G).

Next, we observed the expression patterns of the USP14 protein in several OSCC cell lines and 70 tumor tissue specimens. The results showed that USP14 was highly overexpressed in OSCC cell lines such as HN4, HN6, HN30, and Cal-27, compared to normal HOKs (Fig. 1H). We then used immunohistochemical analysis to further analyze the correlation between USP14 expression and clinicopathological features in 70 patients with OSCC. The results of this analysis showed that USP14 expression in OSCC tumor tissues was significantly higher than that in adjacent normal tissue samples (Fig. 1I), which is consistent with what we found in a previous study [16]. We rated the relative intensity of USP14 expression visually, as weak, moderate, and strong, and used the ratings to construct representative images that are shown in Fig. 1I. Our statistical analysis of these semi-quantitative images indicated that the upregulated expression of USP14 was significantly associated with the AJCC (*P* < 0.001), T (*P* < 0.001) and N stages (*P* = 0.028) of OSCC tumors (Table 1).

**Table 1:** Correlation between USP14 expression and clinicopathological features in patients with OSCC (n = 70)

	Number (70)	USP14 expression		*P* value		Number (70)	USP14 expression		*P* value
		Low (40)	High (30)				Low (40)	High (30)	
Age					Smoking status				
< 60	26	14	12	0.668	Current/former	19	9	10	0.313
≥ 60	44	26	18		Never	51	31	20	
					Alcohol intake				
Gender					Yes	23	11	12	0.271
Male	44	23	21	0.284	No	47	29	18	
Female	26	17	9		AJCC stage				
					I	24	21	3	< 0.001
Location					II	33	16	17	
Tongue	29	15	14	0.123	III	13	3	10	
Buccal	15	12	3		T stage				
Gingiva	9	3	6		T1 + T2	38	30	8	< 0.001
Pharynx oralis	8	3	5		T3 + T4	32	10	22	
Palatal	4	4	0		N stage				
Mouth floor	3	2	1		N0	43	29	14	0.028
Other	2	1	1		N1 + N2	27	11	16	

Taken together, these results strongly suggest that USP14 may facilitate tumorigenesis in OSCC and that its expression also plays an important role in predicting the clinical outcomes of patients with OSCC.

### USP14 promotes proliferation, migration and glycolytic metabolism in oral squamous cell carcinoma cells

**Fig. 2:**
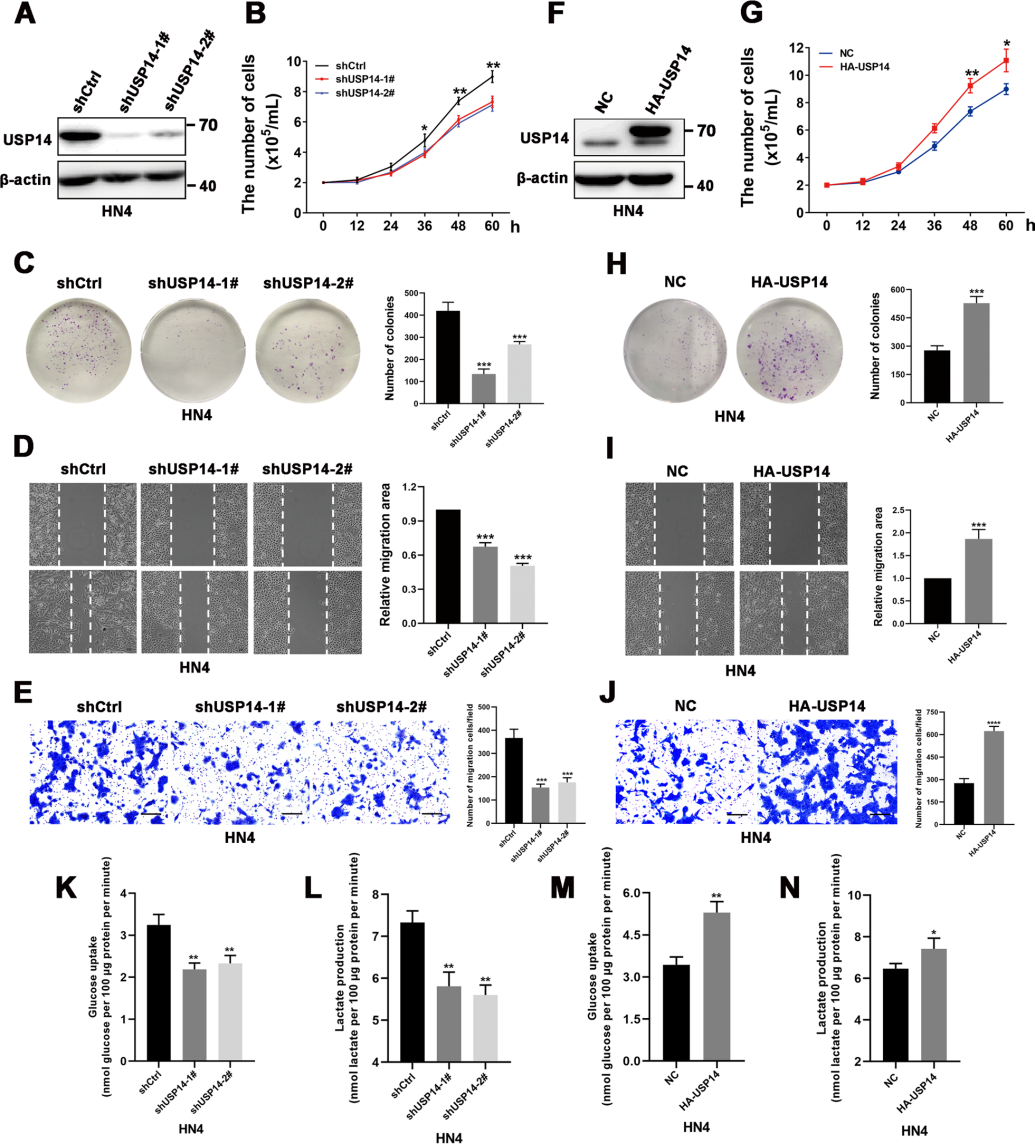
USP14 expression promotes the proliferation, migration, and glycolytic metabolism of oral squamous cell carcinoma cells. **A**, **F** The knockdown or overexpression efficiency of USP14 in HN4 cells was examined by Western blotting. **B**, **G** HN4 cells were transfected with USP14-shRNA or HA-USP14 plasmid. Proliferative ability was determined by cell counting at the indicated time points. Data represent the means ± S.D. of three independent experiments. **C**, **H** HN4 cells were transfected with the indicated plasmids and seeded into 6-well plates at a density of 2000 cells/well. After 10 days, colony formation was detected by crystal violet staining. Data represent the means ± S.D. of three independent experiments. **D**, **I** Wound healing assay was performed on HN4 cells stably transfecting with USP14-shRNA or HA-USP14 plasmid. Images were photographed and analyzed by measurement of the cell-free areas in multiple fields using a service provided by Wimasis. Data represent the means ± S.D. of three independent experiments. **E**, **J** HN4 cells were transfected with the indicated plasmids. Cell migration was investigated using Transwell assay. The numbers of migrated cells per field (mean ± S.D.) from three independent experiments. **K**, **L** Cellular glucose consumption and lactate excretion were detected in HN4 cells with USP14 silencing using glucose uptake assay and lactate colorimetric assay, respectively. Data represent the means ± S.D. of three independent experiments. **M**, **N** Cellular glucose consumption and lactate excretion were detected in HN4 cells with USP14 overexpression. Data represent the means ± S.D. of three independent experiments. *P < 0.05, **P < 0.01, ***P < 0.001

As has been shown previously, USP14 is frequently overexpressed in OSCC and may be involved in tumorigenesis and development. We therefore intended to further explore how USP14 exerts its regulatory function and analyze the corresponding molecular mechanism. By knocking down USP14 expression in OSCC cells using shRNA, we investigated the effects of USP14 on the malignant biological behavior of OSCC cells (Fig. 2A–E, Additional file 1: Fig. S1A, S1C). Compared to the control group, depletion of USP14 in OSCC cells inhibited cell proliferation, colony formation, and migration capacity—all of which represented findings that were consistent with those of our previous study [16]. Conversely, the overexpression of exogenous USP14 in OSCC cells significantly enhanced cell proliferation, colony formation, and migration—all features that promoted the malignant biological behaviors of tumor cells (Fig. 2F–J, Additional file 1: Fig. S1B, S1D). Collectively, these results suggest that USP14 may be closely related to the progression of OSCC.

Glycolysis is well known to be a prominent metabolic hallmark and the main energy source for most tumors, including OSCC [21]. Notably, we observed that high expression levels of USP14 were positively correlated with glycolysis in our TCGA cohort of patients with OSCC (Additional file 1: Fig. S1E). We next intended to investigate whether USP14 also regulates glycolysis in OSCC cells. We observed that depletion of USP14 impaired the glycolytic activity of OSCC cells, as evidenced by significantly reduced levels of glucose uptake and lactate production upon USP14 knockdown (Fig. 2K, L, Additional file 1: Fig. S1F, G). Conversely, both glucose uptake and lactate production were significantly increased in cells that overexpressed USP14 compared to the control group (Fig. 2M, N, Additional file 1: Fig. S1H, I).

Given that USP14 promotes the proliferation, migration, and glycolytic metabolism in OSCC cells, we next assessed whether enhanced glycolysis mediated by USP14 upregulation in HN4 cells is necessary for its tumor-promoting effects. As shown in Additional file 1: Fig. S1J, K, the high capacity of cell proliferation and migration upon USP14 overexpression in HN4 cells were entirely abolished by direct inhibition of glycolysis with the inhibitor 2-deoxyglucose (2-DG). Collectively, these results suggest that the upregulation of USP14 promotes the proliferation and migration in OSCC cells by enhancing their glycolytic capacity, and thus, it may be closely related to the progression of OSCC.

### USP14 interacts with PFKL

**Fig. 3:**
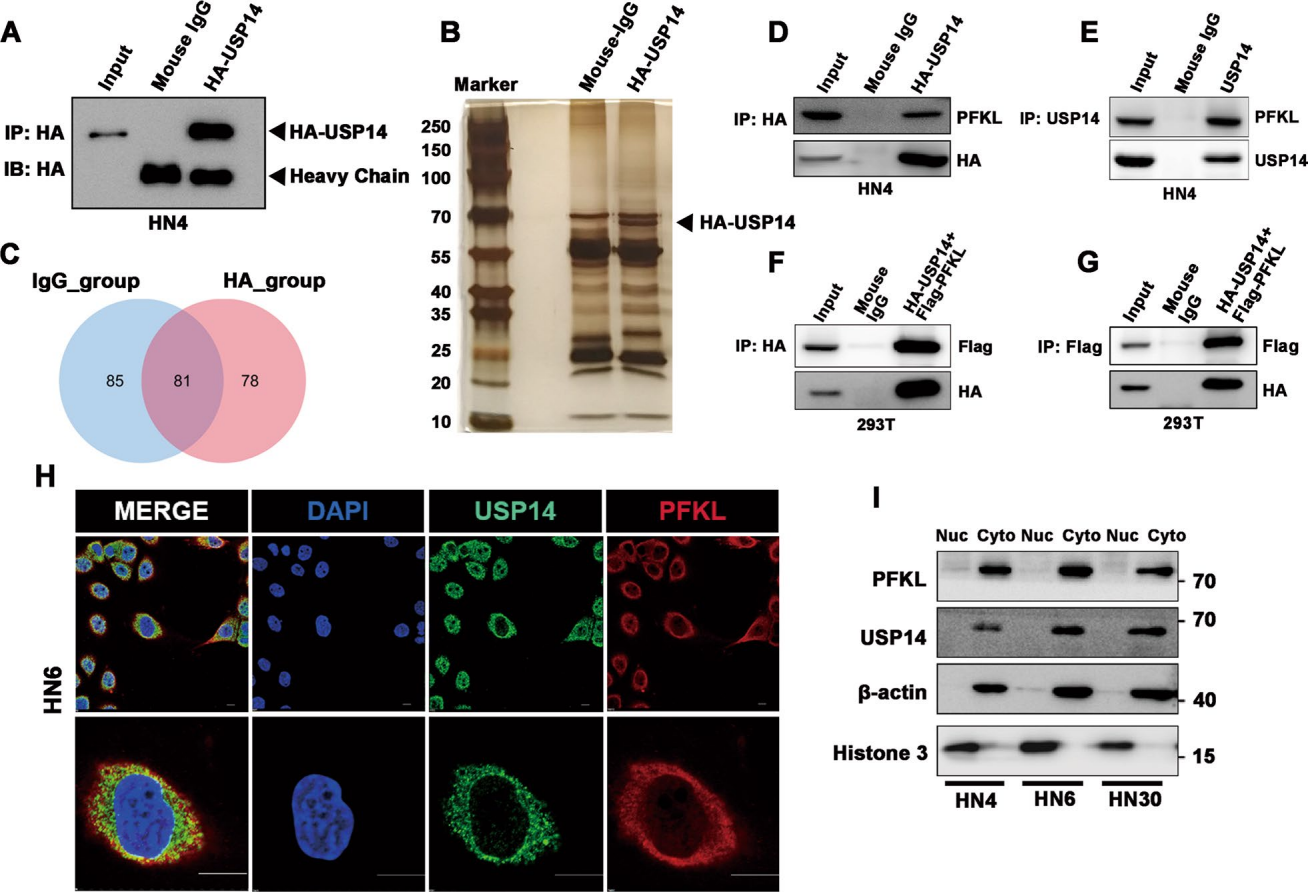
USP14 interacts with PFKL. **A** The exogenous expression of HA-USP14 in the pull-down elution was validated by Western blotting. **B** Representative images of silver-stained protein bands for mass spectrometry (MS) analysis of USP14-interacting proteins. **C** Venn diagram showed the numbers of mouse IgG and HA antibody binding proteins identified by immunoprecipitation coupled with mass spectrometry analysis (IP-MS). **D** Co-immunoprecipitation (Co-IP) analysis of the interactions between exogenous USP14 and endogenous PFKL. Whole-cell lysates from HN4 cells stably expressing HA-USP14 were immunoprecipitated and immunoblotted with antibodies against the indicated proteins. **E** Co-IP analysis of the interactions between endogenous USP14 and PFKL in HN4 cells. **F**, **G** Co-IP analysis of the interactions between exogenous USP14 and PFKL in HEK293T cells. Whole-cell lysates from HEK293T cells stably expressing HA-USP14 and Flag-PFKL were immunoprecipitated and immunoblotted with antibodies against the indicated proteins. **H** Confocal microscopic analysis of USP14 and PFKL subcellular localization. HN6 cells were fixed and immunoblotted with antibodies against the indicated proteins. Representative images from biological triplicate experiments are shown. Scale bar, 10 μM. **I** Nuclear and cytoplasmic proteins in three OSCC cell lines were separated by protein extraction kit and immunoblotted with antibodies against the indicated proteins

As has been reported previously, USP14 regulates the ubiquitination of substrates and maintains protein quality control and homeostasis [22]. However, substrates of USP14 remain poorly characterized, which hinders the understanding of its functional roles. To investigate the mechanism through which USP14 regulates malignant behaviors and glycolysis in OSCC cell lines, we performed an immunoprecipitation assay, followed by mass spectrometry analysis (IP-MS) to identify novel proteins that interact with USP14. We established HN4 cells that stably overexpressed retrovirally-mediated HA-tagged USP14. Lysates from HN4 cells were immunoprecipitated with anti-HA or anti-Mouse IgG antibodies as negative control. The exogenous expression of USP14 in pull-down elution was validated via Western blotting (Fig. 3A). The proteins in the immunoprecipitates were then analyzed via silver staining and mass spectrometry. Silver staining gel showed HA-tagged USP14 enrichment using the anti-HA antibody (indicated by the black arrow). We observed some unique bands in the range of 70–100 kDa, suggesting that proteins with molecular weights in this range are most likely to interact with USP14 (Fig. 3B).

A total of 78 potential target proteins were identified in the HA group after excluding non-specific ones present in both the HA and control group precipitates (Fig. 3C). Consistent with previously reported interacting partners of USP14 [19], several subunits of the 19 S proteasome—including PSMD1, PSMD2, and PSMD3—and 20 S core subunits—including PSMA1, PSMB1, and PSMB6—were identified in HA-USP14 group, but not in the control (Additional file 6: Table S2). Among the list of potential proteins, PFKL, a key rate-limiting enzyme involved in the glycolytic pathway, attracted our attention.

We further verified the interactions between endogenous USP14 and PFKL in HN4 cells, or between exogenous USP14 and PFKL in HEK293T cells, using a co-immunoprecipitation (co-IP) (Fig. 3D–G). To further investigate the association between USP14 and PFKL at the cellular level, immunofluorescence analysis was performed to visualize their distributions. As can be seen in Fig. 3H, these proteins co-localized in the cytoplasm of OSCC cells. The cells were then fractionated into cytoplasmic and nuclear constituents, and the subcellular distributions were detected using Western blotting. The purities of cytoplasmic and nuclear fractions were checked using anti-β-actin and anti-histone 3, respectively. Consistent with the results of immunofluorescence analysis, both USP14 and PFKL showed significant cytoplasmic accumulation (Fig. 3I). Taken together, these results indicate that USP14 physically interacts with PFKL in the cytoplasm of OSCC cells.

### USP14 stabilizes PFKL through deubiquitination

**Fig. 4:**
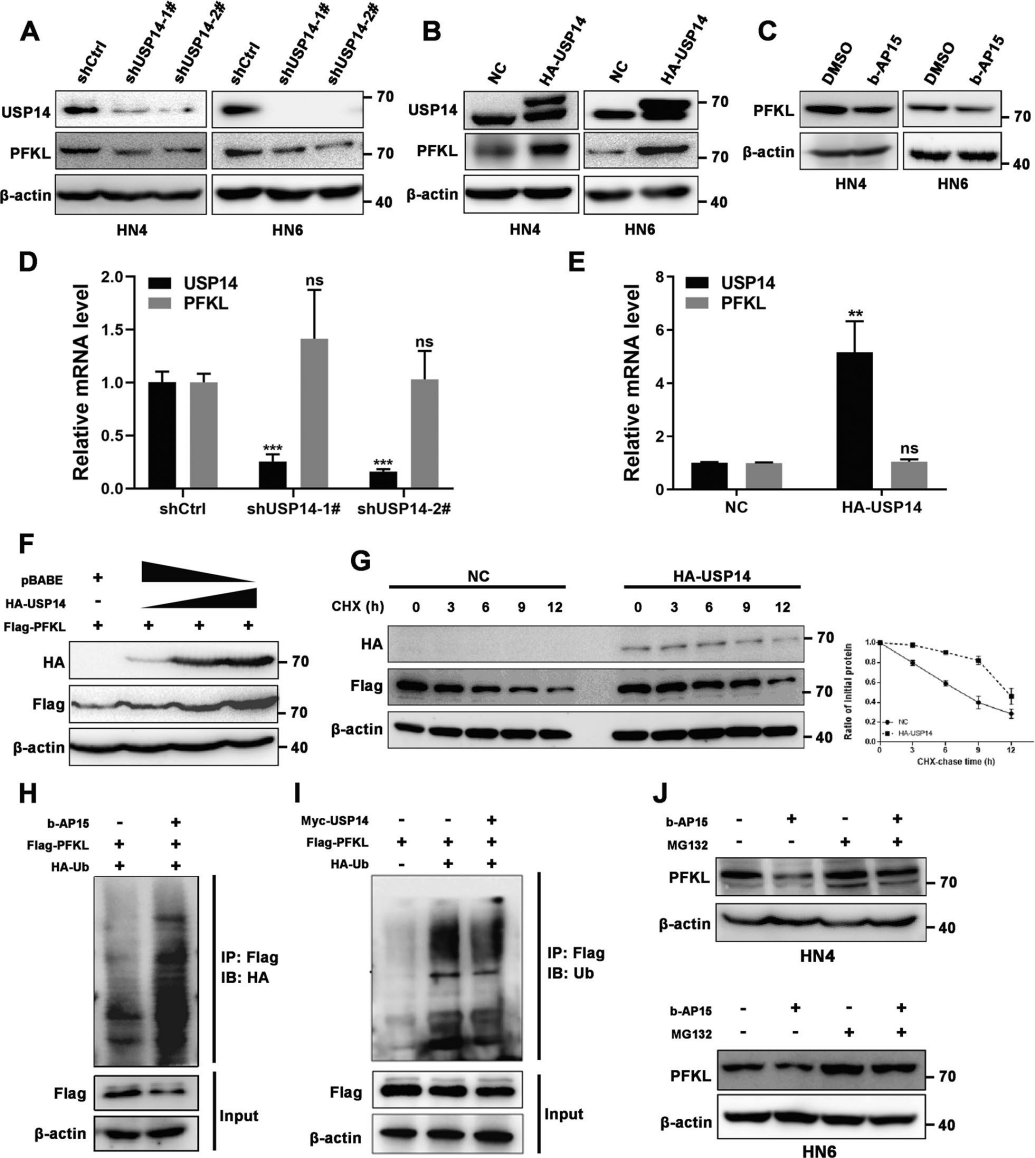
USP14 stabilizes PFKL by promoting its deubiquitination. **A** USP14 was depleted by shRNAs in the OSCC cells and the indicated proteins were examined by Western blotting. **B** USP14 was ectopically overexpressed in the OSCC cells and the indicated proteins were examined by Western blotting. **C** HN4 and HN6 cells were cultured in the absence or presence of b-AP15 (1 μM) for 24 h. Cellular extracts were collected for Western blotting with indicated antibodies. **D**, **E** qRT-PCR analysis of the indicated genes in HN4 cells with USP14 knockdown or overexpression. **F** Increasing amounts of HA-USP14 plasmids were co-transfected with Flag-PFKL plasmid into HEK293T cells and the indicated proteins were examined by Western blotting. **G** HEK293T cells were transfected with HA-USP14 or vector plasmids. A CHX chase experiment was performed and Flag-tagged PFKL protein was determined by Western blotting (left panel). The right panel showcases the relative protein amounts of different groups. Error bars represent ± S.D. of triplicate experiments. **H** HEK293T cells were transfected with HA-Ub and Flag-PFKL plasmids, immunoprecipitation (IP) was performed using Flag antibody. The indicated proteins were examined by Western blotting. **I** HEK293T cells were transfected with HA-Ub, Flag-PFKL and HA-USP14 plasmids, IP was performed using Flag antibody. The indicated proteins were examined by Western blotting. **J** HN4 and HN6 cells were treated with b-AP15 (1 μM) for 24 h and further incubated with or without MG132 (5 μM) for another 5 h. The indicated proteins were examined by Western blotting

It is well known that USP14 impedes the degradation of ubiquitinated proteins by removing ubiquitin chains from its substrates, so we hypothesized that USP14 might stabilize PFKL protein through deubiquitination. To this end, we carried out a series of experiments to explore the regulatory effects and possible mechanisms behind USP14’s effects on the expression of PFKL. The results showed that lentivirus-medicated knockdown of USP14, or the inhibition of USP14’s deubiquitinase activity by b-AP15 (a USP14 inhibitor), could markedly downregulate the protein expression of PFKL (Fig. 4A–C), but not its mRNA expression levels, in HN4 or HN6 cells (Fig. 4D). Exogenous expression of USP14 in these cells, conversely, yielded the opposite results (Fig. 4B–E), suggesting that USP14 might regulate the expression of PFKL at the post-translational level.

Our results also showed that, after HEK293T cells were transfected with 0, 1, 1.5 or 2 μg of the pBABE-HA-USP14 plasmid, the expression of intracellular PFKL increased in a dose-dependent manner (Fig. 4F). Moreover, a cycloheximide (CHX) chase experiment indicated that the co-expression of HA-tagged USP14 could significantly prolong the half-life of exogenous Flag-tagged PFKL compared to the negative control (Fig. 4G). This further supported our original hypothesis that USP14 can stabilize PFKL. Next, we performed in vitro ubiquitination assays to examine whether USP14 could affect the ubiquitination of PFKL. As expected, b-AP15-treated HEK293T cells accumulated ubiquitinated PFKL (Fig. 4H), while the overexpression of USP14 effectively decreased the ubiquitination of PFKL (Fig. 4I). Moreover, the proteasome inhibitor MG132 could attenuate the b-AP15-mediated decrease of PFKL in OSCC cells (Fig. 4J).

Phosphofructokinase includes three isozymes in humans: liver (L), muscle (M), and platelet (P) [8]. We examined whether USP14 also affects the ubiquitination levels of two other isozymes of phosphofructokinase in OSCC. Co-IP showed that USP14 did not physically interact with PFKM or PFKP (Additional file 2: Fig. S2A, B). Next, we performed in vitro ubiquitination assays and observed minimal difference in the ubiquitination level of PFKM or PFKP between HA-USP14 and vector control cells (Additional file 2: Fig. S2C, D). USP14 inhibitor b-AP15 did not significantly affect the ubiquitinated PFKM or PFKP (Additional file 2: Fig. S2E, F). As expected, no obvious expression decrease of PFKM or PFKP was observed during the experiments in HN4 cells receiving b-AP15 or vehicle (Additional file 2: Fig. S2G, H). Collectively, these results demonstrated that USP14 specifically deubiquitinate and stabilize PFKL but has no regulatory effect on the ubiquitination level of PFKM or PFKP.

### PFKL promotes the proliferation and migration of oral squamous cell carcinoma cells by enhancing glucose metabolism

**Fig. 5:**
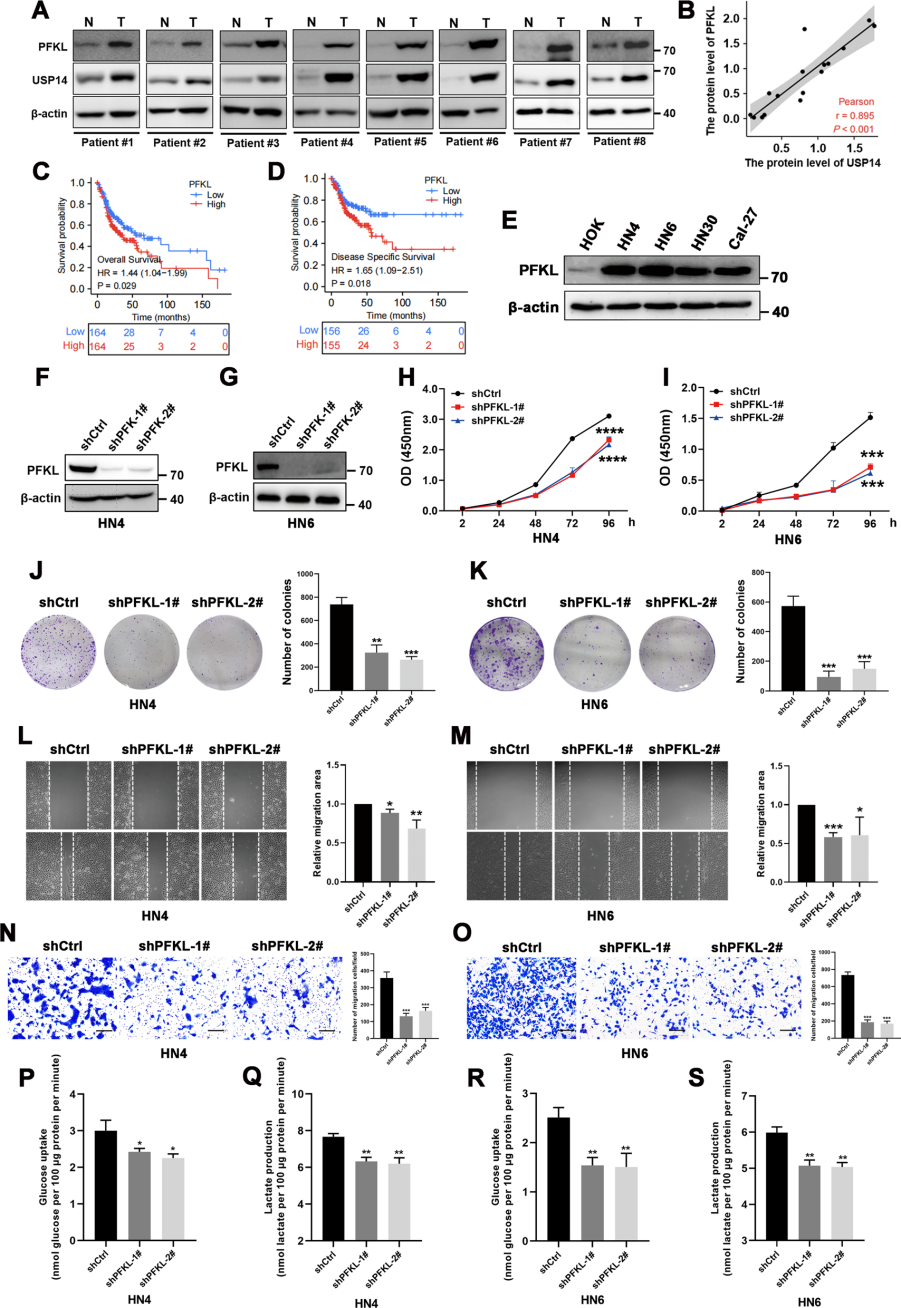
PFKL promotes the proliferation and migration of oral squamous cell carcinoma cells by upregulating glucose metabolism. **A** Western blotting analysis was performed to confirm USP14 expression in OSCC tissues and adjacent normal tissues (n = 8). **B** Pearson analysis was performed to determine the correlations between USP14 and PFKL in OSCC tissues. **C**, **D** Overall survival (OS) and disease specific survival (DSS) based on PFKL expression in OSCC (TCGA). PFKL high (red) group corresponds to the fourth quartile of expression, while PFKL low (blue) group corresponds to the first quartile. **E** The protein levels of PFKL in four OSCC cell lines were compared with a normal oral epithelial cell line HOK by Western blotting analysis. **F**, **G** Western blotting results revealed the knockdown efficiency of PFKL in HN4 and HN6 cells. **H**, **I** HN4 and HN6 cells were transfected with pGIPZ-shPFKL plasmids. Proliferative ability was determined by CCK8 assay at the indicated time points. Data represent the means ± S.D. of three independent experiments. **J**, **K** OSCC cells were transfected with the indicated plasmids and seeded into 6-well plates at a density of 2000 cells/well. After 10 days, colony formation was detected by crystal violet staining. Data represent the means ± S.D. of three independent experiments. **L**, **M** Wound healing assay was performed on OSCC cells stably transfecting with PFKL-shRNA plasmids. Images were photographed and analyzed by measurement of the cell-free areas in multiple fields using a service provided by Wimasis. Data represent the means ± S.D. of three independent experiments. **N**, **O** OSCC cells were transfected with PFKL-shRNA plasmids. Cell migration was investigated using Transwell assay. The numbers of migrated cells per field (mean ± S.D.) from three independent experiments. **P–S** Cellular glucose consumption and lactate excretion were detected in OSCC cells with PFKL silencing using glucose uptake assay and lactate colorimetric assay, respectively. Data represent the means ± S.D. of three independent experiments. *P < 0.05, **P < 0.01, ***P < 0.001, ****P < 0.0001

To explore the clinical relevance of our findings, we further analyzed the expression of USP14 and PFKL in eight pairs of tissue samples from patients with OSCC, using Western blotting. The results showed that both PFKL and USP14 protein expression increased in tumor tissues compared to that in their matched adjacent non-cancerous tissues, which exhibited consistent trends (Fig. 5A, B). We also analyzed the association between PFKL expression and clinical outcomes in patients with OSCC. As shown in Fig. 5C, D, high levels of PFKL expression predicted poor prognoses in both OS (HR = 1.44, *P* = 0.029) and DSS (HR = 1.65, *P* = 0.018). Western blotting analysis also revealed that PFKL protein levels were significantly higher in OSCC cell lines (HN4, HN6, HN30, and Cal-27) than in HOK ones (Fig. 5E).

Next, we constructed stable PFKL-knockdown cells using specific shRNA transfection (Fig. 5F, G), to explore whether the aberrant expression of PFKL contributed to the malignant biological behaviors of OSCC cells. As shown in Fig. 5H–O, the knockdown of PFKL significantly lowered cell proliferation, clone formation, and migration. In view of the critical role of PFKL in tumor glycolysis, we further investigated the effects of its knockdown on glucose uptake and lactate production. As was expected, both were markedly decreased in PFKL-knockdown cells (Fig. 5P–S). Notably, direct blockade of the glycolytic pathway via 2-DG abolished tumor-promoting effects of PFKL (Additional file 3: Fig. S3A–B). Thus, these results demonstrated that the upregulation of PFKL promotes the proliferation and migration of OSCC cells might depend on their enhanced glycolytic capacity.

### USP14 exhibits tumor-promoting roles by enhancing PFKL-mediated glycolytic metabolism

**Fig. 6:**
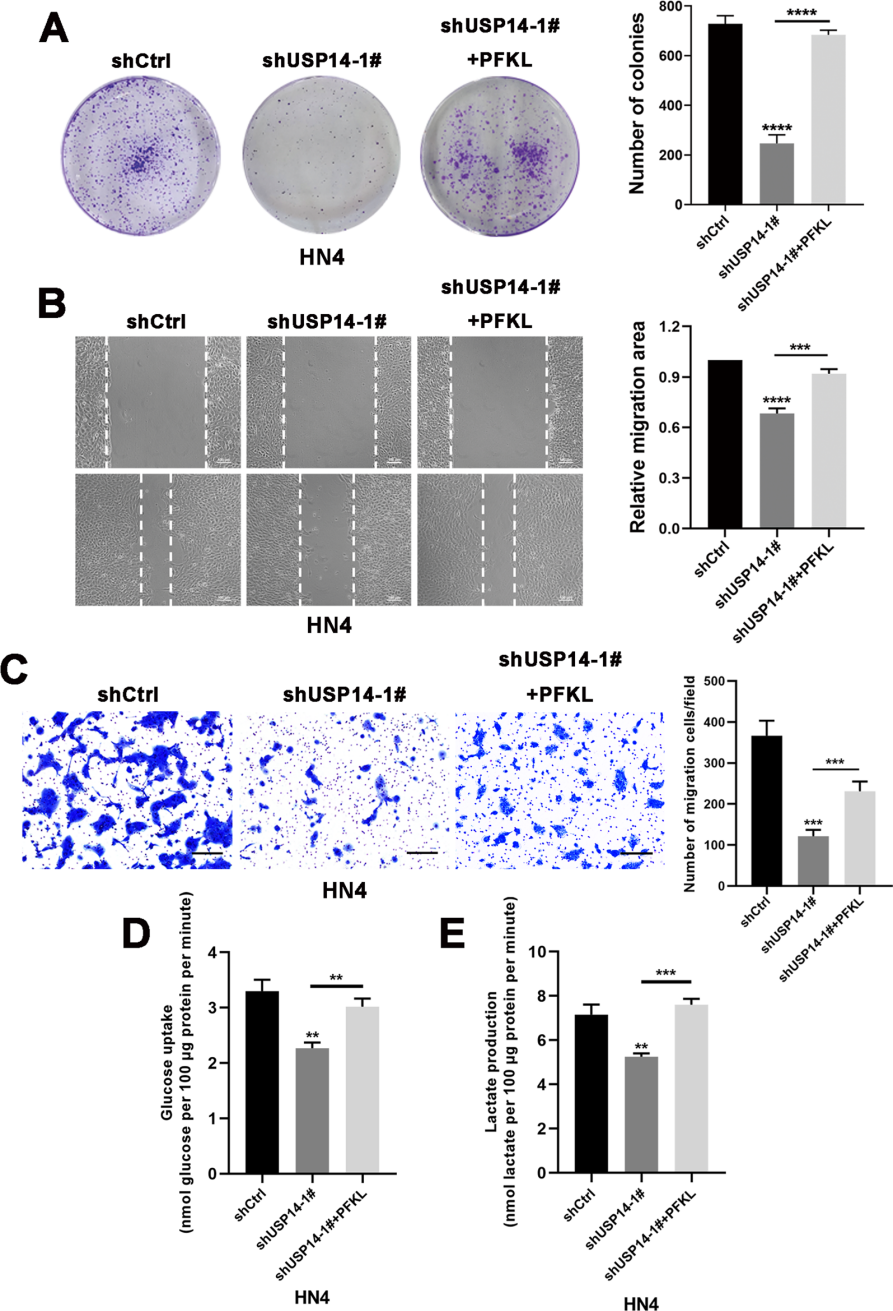
USP14 exhibits tumor-promoting roles by enhancing PFKL-mediated glycolytic metabolism.** A** HN4 cells stably transfecting with either shCtrl, shUSP14-1#, or shUSP14-1# + PFKL were seeded into 6-well plates at a density of 2000 cells/well. After 10 days, colony formation was detected by crystal violet staining. Data represent the means ± S.D. of three independent experiments. **B** Wound healing assay was performed on HN4 cells stably transfecting with the indicated plasmids. Images were photographed and analyzed by measurement of the cell-free areas in multiple fields using a service provided by Wimasis. Data represent the means ± S.D. of three independent experiments. **C** HN4 cells were transfected with the indicated plasmids. Cell migration was investigated using Transwell assay. The numbers of migrated cells per field (mean ± S.D.) from three independent experiments. **D**, **E** Cellular glucose consumption and lactate excretion were detected in HN4 cells stably expressing with the indicated plasmids using glucose uptake assay and lactate colorimetric assay, respectively. Data represent the means ± S.D. of three independent experiments. *P < 0.05, **P < 0.01, ***P < 0.001, ****P < 0.0001

Since we had seen that USP14 could promote glucose metabolism in OSCC cells and stabilize PFKL via deubiquitination, we reasoned that USP14 might exert tumor-promoting effects by upregulating the expression of PFKL and enhancing PFKL-mediated glycolysis in OSCC. To test this, we performed a series of functional rescue experiments. First, we knocked down endogenous USP14 in OSCC cells using transfection with specific shRNAs and then rescued PFKL via ectopic expression. As shown in Fig. 6 and Additional file 4: Fig. S4, the forced expression of PFKL in USP14-knockdown OSCC cells could partially restore clone formation, migration, glucose uptake, and lactate production. Collectively, these results provide evidence that USP14 may function as an upstream regulator of PFKL by upregulating its expression, to enhance PFKL-mediated glycolysis in OSCC.

### The oncogenic properties of USP14 in oral squamous cell carcinoma xenograft mouse models

**Fig. 7:**
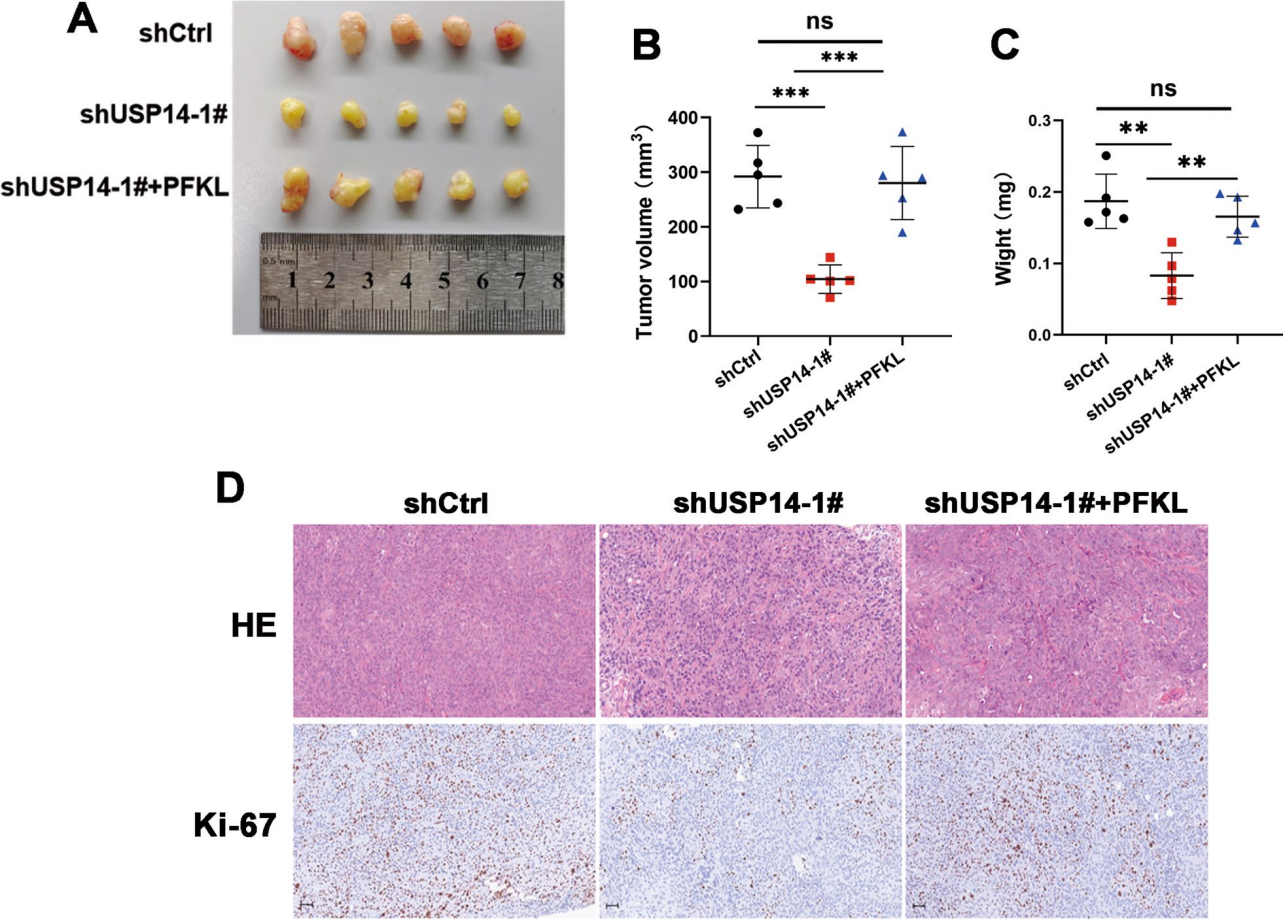
Oncogenic properties of USP14 in oral squamous cell carcinoma xenograft mouse models. **A–C** A total of 2 × 10^6^ HN6 cells stably transfecting with either shCtrl, shUSP14-1#, or shUSP14-1# + PFKL were subcutaneously injected into nude mice to establish an OSCC xenograft mouse model. After 3 weeks, tumors from 15 of the mice were extracted and photographed. Tumor images (**A**), tumor volume (**B**) and weight (**C**) were then assessed. **D** Expression pattern of Ki-67 was examined by immunohistochemistry analysis in the xenograft tumors of each group. Original magnification, ×100. **P < 0.01, ***P < 0.001

We subcutaneously injected HN6 cells into the right flanks of nude mice, to establish an OSCC xenograft mouse model. This was then used to study the tumor-promoting effect of USP14 in vivo. Our results showed that, compared to the control group, the stable knockdown of USP14 significantly decreased the tumor volumes (Fig. 7A, B) and weights (Fig. 7C). Notably, the overexpression of PFKL in USP14-knockdown HN6 cells could significantly weaken the tumor-suppressing effects induced by depletion of USP14 (Fig. 7A–C). We then assessed the expression of the proliferation marker Ki-67 in the tumor tissues via immunohistochemical staining and found that it was significantly reduced in USP14-depleted tumors. However, this was again reversed by overexpressing PFKL (Fig. 7D). These results suggest that USP14 can also drive the progression of OSCC by regulating the expression of PFKL in vivo.

## Discussion

We previously reported that USP14 was aberrantly upregulated in OSCC; however, the underlying mechanisms behind this were not addressed [16]. In this study, we further demonstrated that the aberrant upregulation of USP14 was closely correlated with adverse clinicopathological features and poor prognosis in patients with OSCC. Notably, we proved for the first time that USP14 could regulate the stability of PFKL in OSCC cells, thereby enhancing their glycolytic capacity and contributing to both cell proliferation and migration.

Aerobic glycolysis is a well-known hallmark of tumorigenesis. Recently, the role of ubiquitination and deubiquitination in the regulation of aerobic glycolysis has been reviewed extensively, highlighting potential approaches to reversing the Warburg effect by targeting post-translational modification [23]. For example, hexokinase 2 (HK2) can be ubiquitinated and deubiquitinated by TRAF6 [24] and COP9 signalosome 5 (CSN5), respectively [25]. Pyruvate kinase M2 (PKM2), the final glycolytic rate-limiting enzyme, was reported to interact with E3 ligases, including Parkin, the carboxyl terminus of heat shock protein 70-interacting protein (CHIP), tripartite motif containing 58 (TRIM58), and the deubiquitinating enzyme (DUB) USP20 [26–29]. USP14 is a major proteasome-associated DUB that belongs to the subfamily of ubiquitin-specific proteases (USPs). Other than its role in the proteasome, USP14 has been reported to be involved in the regulation of lipid and carbohydrate metabolism [30]. However, the biological functions of USP14 in cancer-related glycolysis have not been explored extensively. To the best of our knowledge, our findings are the first to reveal a novel function of USP14 in the regulation of OSCC glycolysis. USP14 facilitates glycolytic activity in OSCC cells, thereby promoting tumor growth both in vivo and in vitro. This suggests, a broad regulatory role of USP14 in cellular metabolism.

PFKL is a master regulator of glycolysis. Previous studies have shown that the ubiquitin E3 ligase A20 mediates the ubiquitination of PFKL, which marks it for degradation—leading to the suppression of tumor growth in liver cancer cells [31]. However, the deubiquitination modification of PFK1 has rarely been reported on in the literature thus far. In this study, we demonstrated that USP14 can deubiquitinate and stabilize PFKL, thereby influencing glucose metabolism in OSCC cells. While a number of studies have addressed the role of glycolysis in tumor cells, few of these have focused on PFKL and its consequences for tumor progression. Our findings indicate that PFKL is highly expressed in OSCC cells and tissues and supports OSCC cell proliferation, clone formation, and migration in vitro, by increasing glycolytic metabolism. Notably, our functional rescue experiments suggested that PFKL is a key factor that contributes to USP14’s tumor-promoting roles in OSCC. Thus, targeting the USP14/PFKL axis may represent a novel strategy for fighting OSCC. Considering that glycolysis generates a highly acidic, nutrient-deficient, and hypoxic tumor microenvironment (TME), which further alters the infiltration of immune cells and local tumor-specific immune responses [32, 33], the direct effect of USP14 on immune responses in the TME is an interesting question that merits further investigation in future studies.

**Fig. 8:**
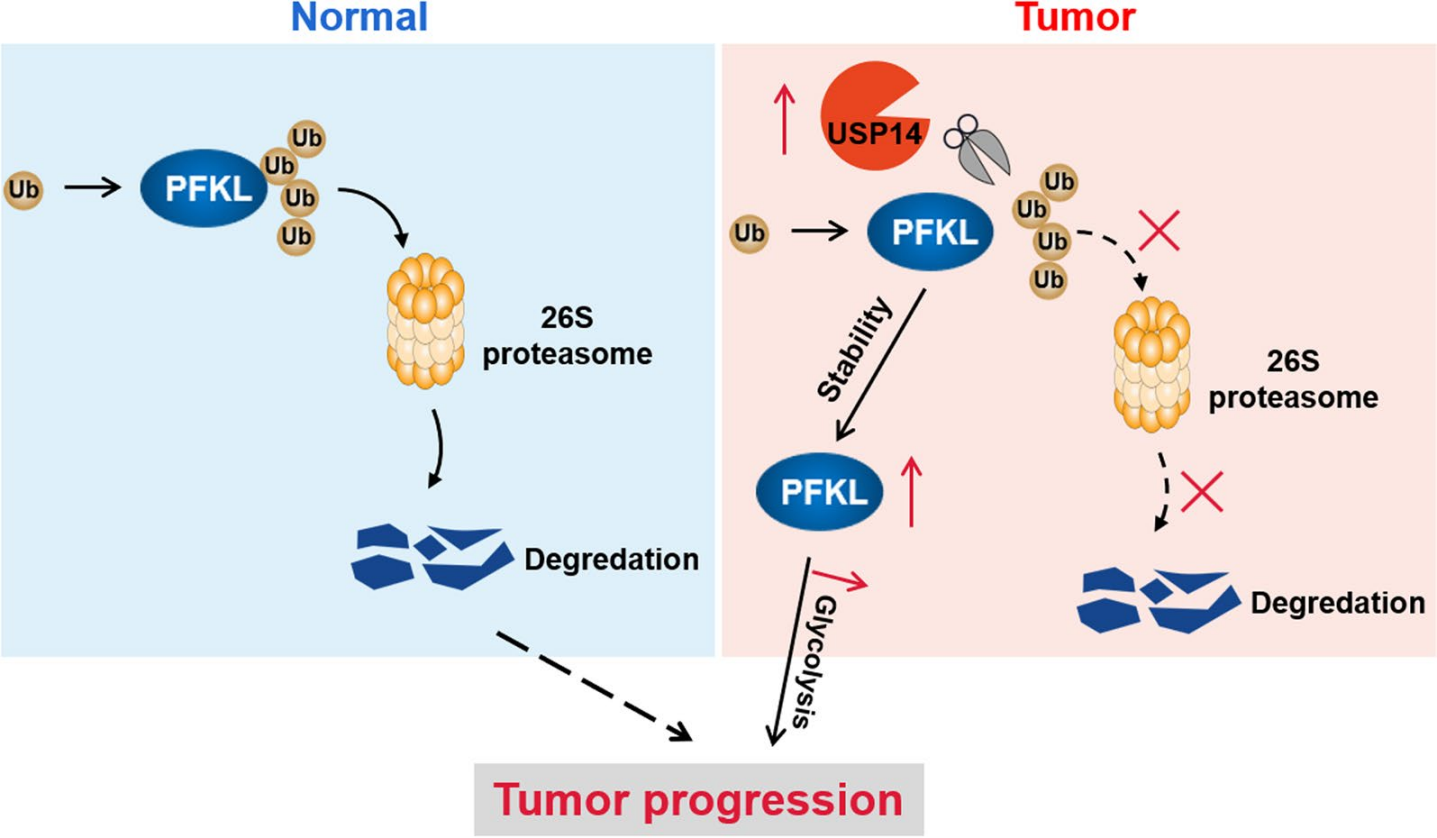
Diagram illustrating the USP14-mediated deubiquitylation and stabilization of PFKL

We demonstrated that USP14 specifically interacts with PFKL and enhances its stability through deubiquitination in OSCC cells, which in turn enhances PFKL-mediated glycolytic metabolism, ultimately promoting the proliferation, migration, and tumorigenesis of tumor cells (Fig. 8). Our findings provide novel insights into the tumor-promoting role of USP14 and establish mechanistic foundations for USP14-targeted therapies.

### Supplementary Information

**Additional file 1: Figure S1.** USP14 expression promotes proliferation, migration, and glycolytic metabolism of oral squamous cell carcinoma cells. **A**, **B**. Cell migration was investigated after HN6 cells were transfected with pGIPZ-shUSP14 or pBABE-HA-USP14 plasmids using Transwell assay. The numbers of migrated cells per field (mean ± S.D.) from three independent experiments. **C**, **D**. The EMT-related markers in USP14-knocked down or USP14-overexpressing cells were examined using Western blotting. **E**. Glycolysis scores based on USP14 expression in OSCC (TCGA). USP14 high (red) group corresponds to the fourth quartile of expression, while USP14 low (blue) group corresponds to the first quartile. **F**, **G**. Cellular glucose consumption and lactate excretion were detected in HN6 cells with USP14 silencing using glucose uptake assay and lactate colorimetric assay, respectively. Error bars represent ± S.D. of triplicate experiments. **H**, **I**. Cellular glucose consumption and lactate excretion were detected in HN6 cells with USP14 overexpression. Error bars represent ± S.D. of triplicate experiments. **J**. The control group, the USP14 overexpression group, and the USP14 overexpression group supplemented with 2-DG (5 mM) from HN4 cells were undergone CCK8 assay. Data represent the means ± S.D. of three independent experiments. **K.** HN4 cells were transfected with pBABE-HA-USP14 plasmids. Cell migration was investigated in the presence or absence of 2-DG using Transwell assay. The numbers of migrated cells per field (mean ± S.D.) from three independent experiments. *P < 0.05, **P < 0.01, ***P < 0.001, ****P < 0.0001.

**Additional file 2: Figure S2.** USP14 has no regulatory effect on the ubiquitination level of PFKM or PFKP. **A**, **B**. Co-immunoprecipitation (Co-IP) was performed using USP14 (A) or HA (B) antibody. The indicated proteins were examined by Western blotting. **C**, **D**. HN4 cells were transfected with HA-USP14, immunoprecipitation (IP) was performed using PFKM (C) or PFKP (D) antibody. The indicated proteins were examined by Western blotting. **E**, **F**. HN4 cells were treated with USP14 inhibitor b-AP15 (1 μM), IP was performed using PFKM (E) or PFKP (F) antibody. The indicated proteins were examined by Western blotting. **G**, **H**. HN4 cells were treated with b-AP15 in the presence or absence of MG132 (5 μM) and the indicated proteins were examined by Western blotting.

**Additional file 3: Figure S3.** Direct inhibition of glycolysis with 2-deoxy-d-glucose (2-DG) abolished tumor-promoting effects of PFKL. **A**. The control group, the PFKL overexpression group, and the PFKL overexpression group supplemented with 2-DG (5 mM) from HN4 cells were undergone CCK8 assay. Data represent the means ± S.D. of three independent experiments. **B**. HN4 cells were transfected with pBABE-Flag-PFKL plasmids. Cell migration was investigated in the presence or absence of 2-DG using Transwell assay. The numbers of migrated cells per field (mean ± S.D.) from three independent experiments. **P < 0.01, ****P < 0.0001.

**Additional file 4: Figure S4.** USP14 exhibits tumor-promoting roles through enhancing PFKL-mediated glycolytic metabolism. **A.** Cell migration was investigated after HN6 cells were transfected with specific shRNAs and then rescued with ectopic expression of PFKL using Transwell assay. The numbers of migrated cells per field (mean ± S.D.) from three independent experiments. ***P < 0.001.

**Additional file 5: Table S1.** Search parameters of MASCOT software.

**Additional file 6: Table S2.** Mass spectrometry analysis report.

## Data Availability

Publicly available datasets were analyzed in this study. These data can be found at https://portal.gdc.cancer.gov/.
