# Supplementary figures and images for: Ubiquitin-specific protease 14 targets PFKL-mediated glycolysis to promote the proliferation and migration of oral squamous cell carcinoma
Source: J Transl Med. 2024 Feb 22;22:193. doi: 10.1186/s12967-024-04943-z (PMC10885370; doi:10.1186/s12967-024-04943-z)

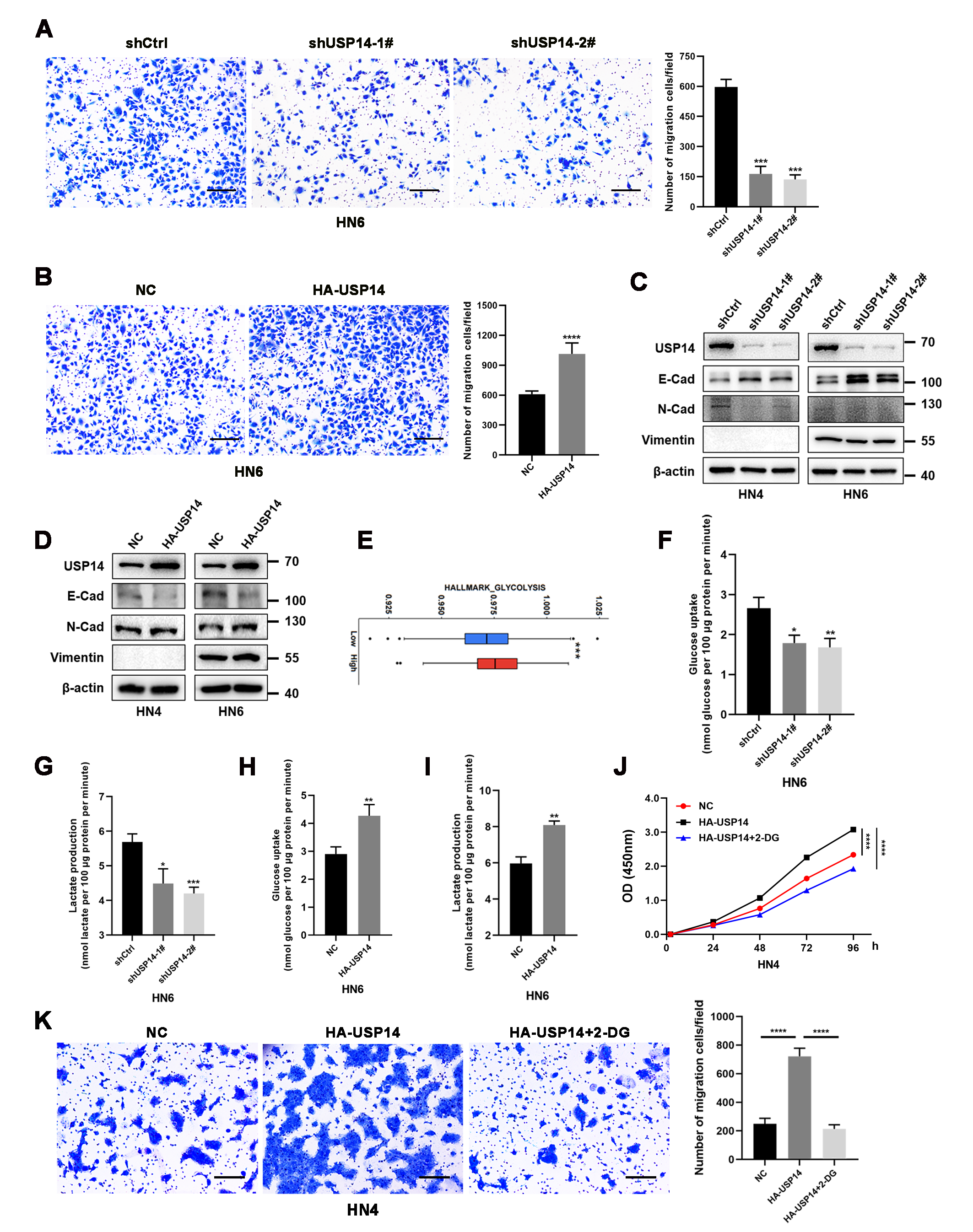

Supplement: Supplementary file 1 — Additional file 1: Figure S1. USP14 expression promotes proliferation, migration, and glycolytic metabolism of oral squamous cell carcinoma cells. A, B. Cell migration was investigated after HN6 cells were transfected with pGIPZ-shUSP14 or pBABE-HA-USP14 plasmids using Transwell assay. The numbers of migrated cells per field (mean ± S.D.) from three independent experiments. C, D. The EMT-related markers in USP14-knocked down or USP14-overexpressing cells were examined using Western blotting. E. Glycolysis scores based on USP14 expression in OSCC (TCGA). USP14 high (red) group corresponds to the fourth quartile of expression, while USP14 low (blue) group corresponds to the first quartile. F, G. Cellular glucose consumption and lactate excretion were detected in HN6 cells with USP14 silencing using glucose uptake assay and lactate colorimetric assay, respectively. Error bars represent ± S.D. of triplicate experiments. H, I. Cellular glucose consumption and lactate excretion were detected in HN6 cells with USP14 overexpression. Error bars represent ± S.D. of triplicate experiments. J. The control group, the USP14 overexpression group, and the USP14 overexpression group supplemented with 2-DG (5 mM) from HN4 cells were undergone CCK8 assay. Data represent the means ± S.D. of three independent experiments. K. HN4 cells were transfected with pBABE-HA-USP14 plasmids. Cell migration was investigated in the presence or absence of 2-DG using Transwell assay. The numbers of migrated cells per field (mean ± S.D.) from three independent experiments. *P < 0.05, **P < 0.01, ***P < 0.001, ****P < 0.0001. [file 12967_2024_4943_MOESM1_ESM.tif]

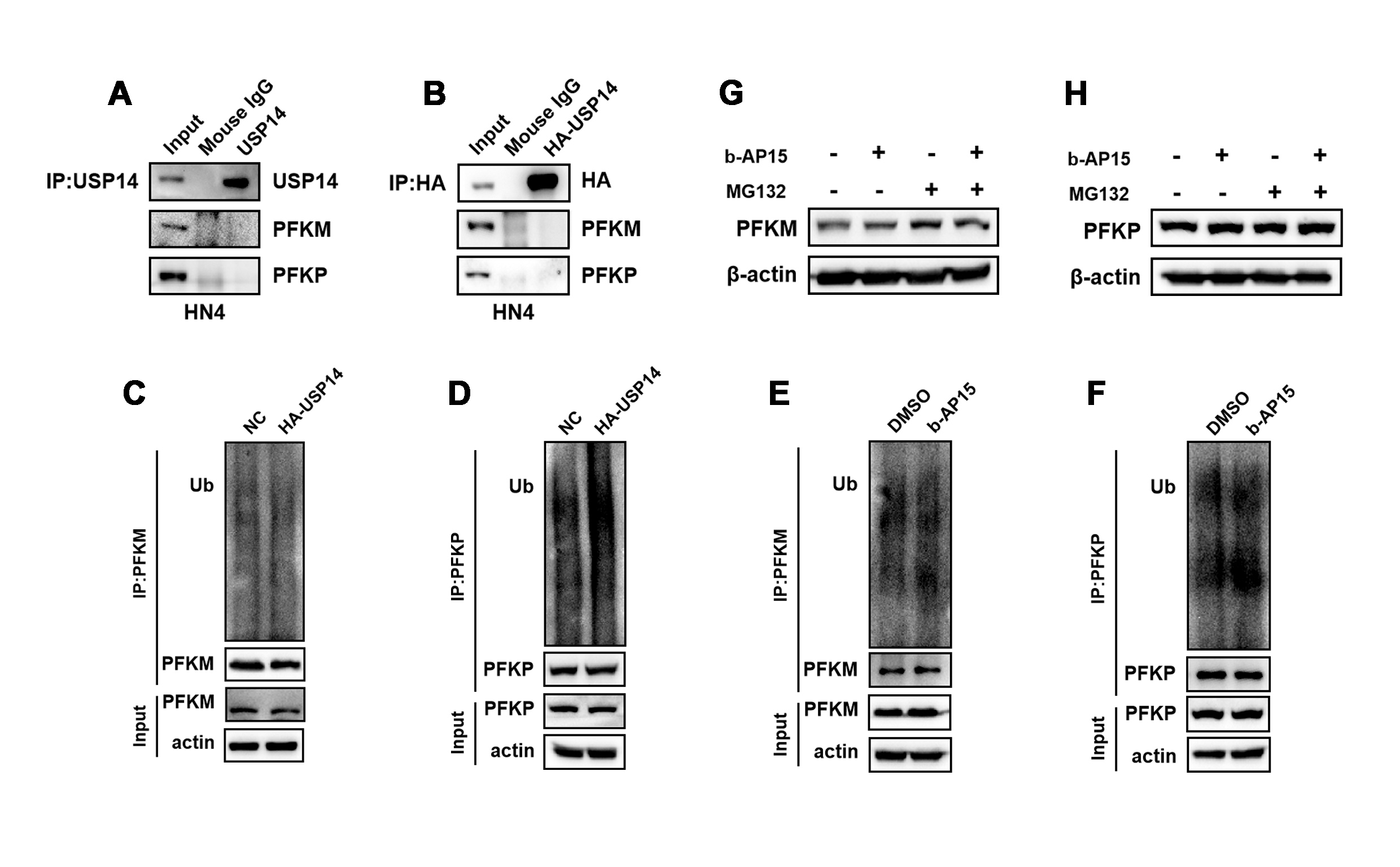

Supplement: Supplementary file 2 — Additional file 2: Figure S2. USP14 has no regulatory effect on the ubiquitination level of PFKM or PFKP. A, B. Co-immunoprecipitation (Co-IP) was performed using USP14 (A) or HA (B) antibody. The indicated proteins were examined by Western blotting. C, D. HN4 cells were transfected with HA-USP14, immunoprecipitation (IP) was performed using PFKM (C) or PFKP (D) antibody. The indicated proteins were examined by Western blotting. E, F. HN4 cells were treated with USP14 inhibitor b-AP15 (1 μM), IP was performed using PFKM (E) or PFKP (F) antibody. The indicated proteins were examined by Western blotting. G, H. HN4 cells were treated with b-AP15 in the presence or absence of MG132 (5 μM) and the indicated proteins were examined by Western blotting. [file 12967_2024_4943_MOESM2_ESM.tif]

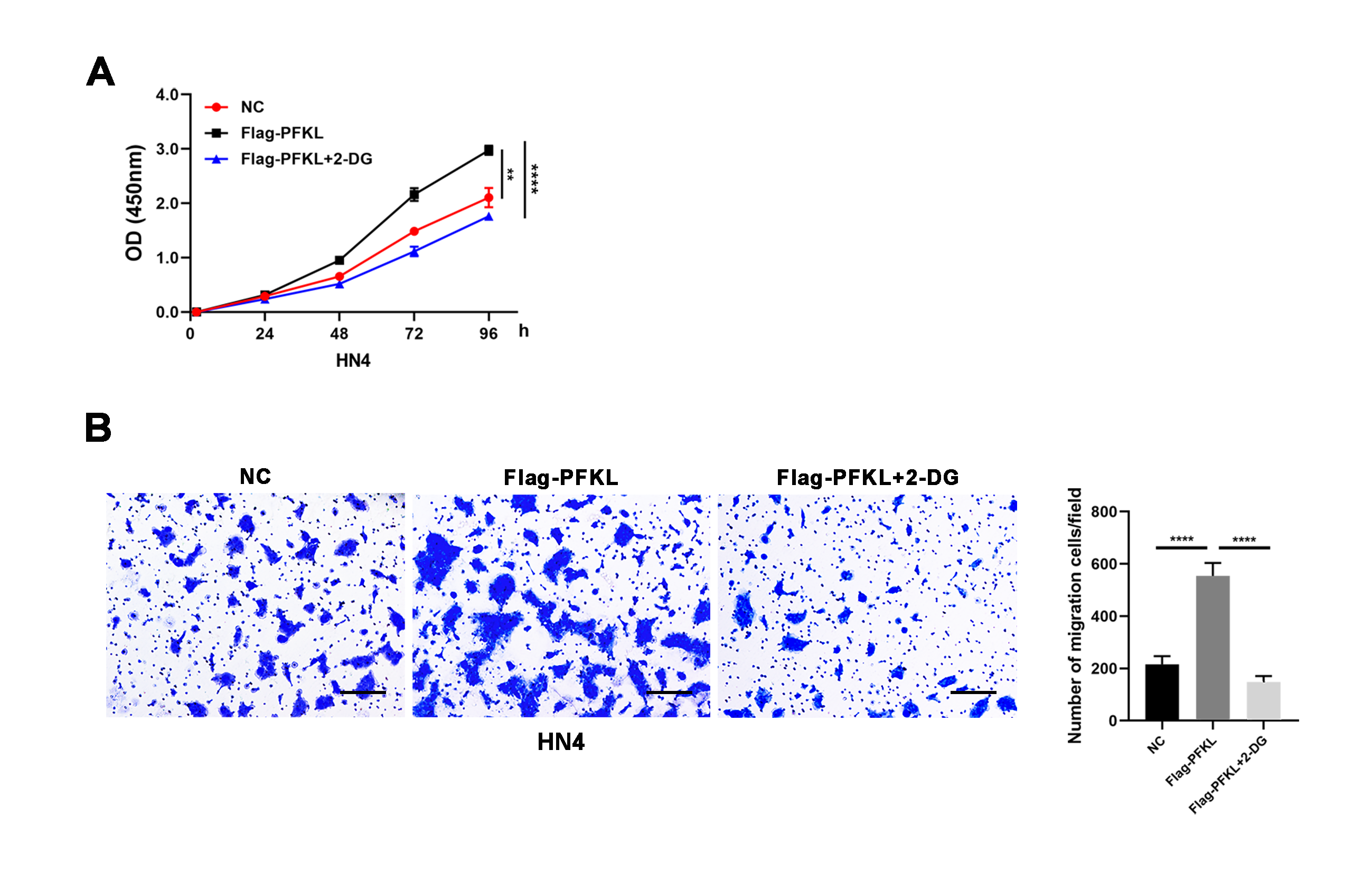

Supplement: Supplementary file 3 — Additional file 3: Figure S3. Direct inhibition of glycolysis with 2-deoxy-d-glucose (2-DG) abolished tumor-promoting effects of PFKL. A. The control group, the PFKL overexpression group, and the PFKL overexpression group supplemented with 2-DG (5 mM) from HN4 cells were undergone CCK8 assay. Data represent the means ± S.D. of three independent experiments. B. HN4 cells were transfected with pBABE-Flag-PFKL plasmids. Cell migration was investigated in the presence or absence of 2-DG using Transwell assay. The numbers of migrated cells per field (mean ± S.D.) from three independent experiments. **P < 0.01, ****P < 0.0001. [file 12967_2024_4943_MOESM3_ESM.tif]

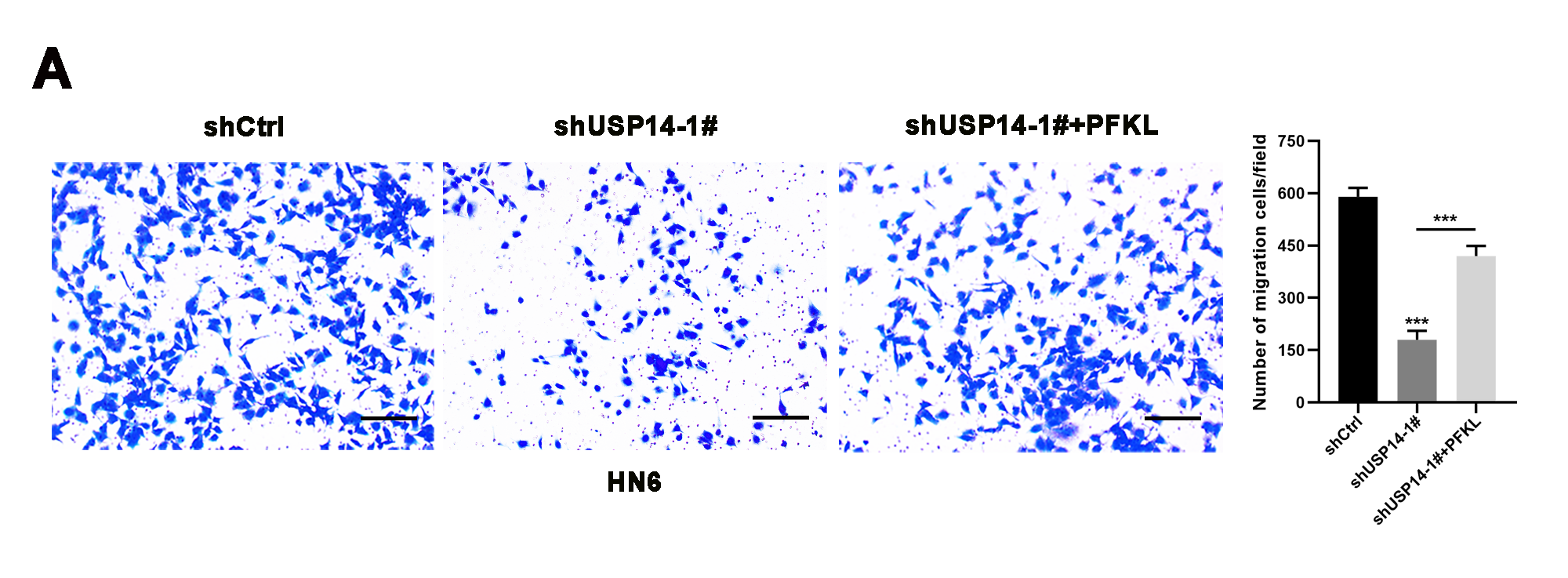

Supplement: Supplementary file 4 — Additional file 4: Figure S4. USP14 exhibits tumor-promoting roles through enhancing PFKL-mediated glycolytic metabolism. A. Cell migration was investigated after HN6 cells were transfected with specific shRNAs and then rescued with ectopic expression of PFKL using Transwell assay. The numbers of migrated cells per field (mean ± S.D.) from three independent experiments. ***P < 0.001. [file 12967_2024_4943_MOESM4_ESM.tif]
