# Supplementary material for: Ubiquitin-specific protease 14 targets PFKL-mediated glycolysis to promote the proliferation and migration of oral squamous cell carcinoma
Source: J Transl Med. 2024 Feb 22;22:193. doi: 10.1186/s12967-024-04943-z (PMC10885370; doi:10.1186/s12967-024-04943-z)
Supplement: Supplementary file 5 — Additional file 5: Table S1. Search parameters of MASCOT software. [file 12967_2024_4943_MOESM5_ESM.docx]

|  | **Table S1. Search parameters of MASCOT software** | |
| --- | --- | --- |
|  | Parameters | Name |
|  | Type of search | MS/MS Ion Search |
|  | Enzyme | Trypsin |
|  | Fixed modifications | Carbamidomethyl (C) |
|  | Variable modifications | Oxidation (M) Acetyl (Protein N-term) |
|  | Mass values | Monoisotopic |
|  | Protein mass | Unrestricted |
|  | Peptide mass tolerance | ± 10 ppm |
|  | Fragment mass tolerance | ± 0.05 Da |
|  | Max missed cleavages | 1 |
|  | Instrument type | ESI-FTICR |
